# Thymosin Beta 4 Is Involved in the Development of Electroacupuncture Tolerance

**DOI:** 10.3389/fncel.2019.00075

**Published:** 2019-03-26

**Authors:** Juan Wan, Yi Ding, Sha Nan, Qiulin Zhang, Jinrui Sun, Chuanguang Suo, Mingxing Ding

**Affiliations:** College of Veterinary Medicine, Huazhong Agricultural University, Wuhan, China

**Keywords:** electroacupuncture tolerance (EAT), opioid peptides, anti-opioid peptides, mu opioid receptor (MOR), CCK B type receptor (CCKBR)

## Abstract

**Background:** Electroacupuncture (EA) tolerance, a negative therapeutic effect, is a gradual decline in antinociception because of its repeated or prolonged use. This study aims to explore the role of thymosin beta 4 (Tβ4), having neuro-protection properties, in EA tolerance (EAT).

**Methods:** Rats were treated with EA once daily for eight consecutive days to establish EAT, effect of Tβ4 on the development of EAT was determined through microinjection of Tβ4 antibody and siRNA into the cerebroventricle. The mRNA and protein expression profiles of Tβ4, opioid peptides (enkephalin, dynorphin and endorphin), and anti-opioid peptides (cholecystokinin octapeptide, CCK-8 and orphanin FQ, OFQ), and mu opioid receptor (MOR) and CCK B receptor (CCKBR) in the brain areas (hypothalamus, thalamus, cortex, midbrain and medulla) were characterized after Tβ4 siRNA was administered.

**Results:** Tβ4 levels were increased at day 1, 4, and 8 and negatively correlated with the changes of tail flick latency in all areas. Tβ4 antibody and siRNA postponed EAT. Tβ4 siRNA caused decreased Tβ4 levels in all areas, which resulted in increased enkephalin, dynorphin, endorphin and MOR levels in most measured areas during repeated EA, but unchanged OFQ, CCK-8, and CCKBR levels in most measured areas. Tβ4 levels were negatively correlated with enkephalin, dynorphin, endorphin, or MOR levels in all areas except medulla, while positively correlated with OFQ and CCK-8 levels in some areas.

**Conclusion:** These results confirmed Tβ4 facilitates EAT probably through negatively changing endogenous opioid peptides and their receptors and positively influencing anti-opioid peptides in the central nervous system.

## Introduction

Electroacupuncture (EA), derived from traditional hand acupuncture, has been widely used for treating various pains with few side effects (Xum et al., 2009; Zeng et al., 2016; Zhao et al., 2017). EA stimulation for a short time (10–40 min) can induce analgesic effect (Ulett et al., 1998; Qiu et al., 2015; Hu et al., 2016). However, prolonged or repeated EA stimulations would attenuate and finally nullify analgesic effect, which is termed “EA tolerance” (EAT) (Han et al., 1979, 1981b).

Because the tolerance to EA results in the decrease or even loss of its treatment effects, it has attracted more attention from practitioners and researchers. To explore EAT mechanism, some studies focus on the roles of analgesic and anti-analgesic neuromodulators in the central nerve system (CNS). It has been verified that EA produces analgesic effect through the release of endogenous opioid peptides (EOP) (Chen and Han, 1992b; Han, 2003). Tang et al. (1979) found repeated EA induced the higher levels of EOP in EAT rats. Further studies demonstrated that EA induced the release of anti-opioid substances including cholecystokinin octapeptide (CCK-8) and orphanin FQ (OFQ) (Bian et al., 1993; Tian et al., 1998) while it provoked the release of opioid peptides. The increase in anti-opioid peptides is believed to be due to the feedback regulation of opioid peptides. Tang et al. (1997) microinjected CCK antisense RNA into the lateral cerebral ventricle of rats to block the CCK gene expression, and found the development of tolerance elicited by prolonged EA stimulation was delayed. Tian and Han (2000) reported that intracerebroventricularly injecting OFQ antibody partially reversed tolerance to chronic EA. These studies indicate that the tipping of the balance to anti-opioid peptides may attenuate EA analgesia and contribute to EAT.

Opioid peptides or anti-opioid peptides exert analgesic or anti-analgesic effect through binding to their corresponding receptors. Therefore, some researchers have paid attention to the roles of these receptors in EAT. Ni et al. (1987) developed EAT and found that the opioid receptors decreased in the brain of rats. Dong et al. (1998) also found the decreased level of opioid receptors in the midbrain and striatum of rats after repeated application of EA. Huang et al. (2007) reported that CCK B type receptor (CCKBR) antagonist (L365, 260) can potentiate EA-induced analgesia and reverse chronic EAT. These findings showed that EAT was related to a change in opioid and anti-opioid receptors levels. However, the specific mechanism deserves further investigation.

Recently, Hu et al. (2018) found thymosin beta 4 (Tβ4) gene differentially expressed in the periaqueductal gray at 4 h in EA-treated goats. Tβ4, a 44-amino acid pleiotropic polypeptide, has important roles in neurobiological processes, including neurogenesis, neuronal developing, metabolism plasticity, and apoptosis (Sun and Kim, 2007; Chopp and Zheng, 2015). Recent studies reported that Tβ4 had a neuro-protective effect (Xiong et al., 2012a,b). EAT induced by prolonged or repeated EA stimulation can be considered as a neuro-protection response. Therefore, there may be a potential association between Tβ4 and EAT, which is worthy being studied.

In the present study, rats were stimulated with EA for 30 min once daily for eight consecutive days to establish EAT. Effect of Tβ4 on the development of EAT was determined through microinjection of Tβ4 antibody and siRNA into the cerebroventricle. The expression profiles of Tβ4, opioid peptides (endorphin, encephalin, and dynorphin), anti-opioid peptides (CCK-8 and OFQ) and related receptors (mu receptor and CCKBR) in the brain were characterized at mRNA and protein levels after Tβ4 siRNA was intracerebroventricularly administered to determine effects of Tβ4 on these proalgesic or analgesic substances and to further explore the role of Tβ4 in EAT.

## Materials and Methods

### Animals

The study was conducted under the guidelines approved by Institutional Animal Care and Use Committee of Huazhong Agricultural University, Wuhan, China and adhered to the guidelines of the Committee for Research and Ethical Issues of the International Association for the Study of Pain.

Female Sprague–Dawley rats (No. 42000600024665) weighing 200–220 g were purchased from the laboratory animal center of Huazhong Agricultural University. Rats were housed six per cage with food pellets and water *ad libitum*. One week was allowed for adaptation to the surroundings.

### Experiment Design

To explore the dynamic expression of Tβ4 in the brain of rats induced by repeated EA, thirty rats were randomly divided into sham group (*n* = 6) and EA group (*n* = 24). The rats in EA group were treated with EA once per day for 8 days consecutively. The rats in sham group were treated in the same manner as the rats in EA group, but without electricity. The tail-flick latency (TFL) was detected everyday immediately before and after EA, respectively, and the change rates of TFL were calculated. Six rats from EA group at day 0 (before EA), 1, 4, and 8, respectively, were euthanized.

To investigate the effect of Tβ4 neutralizing antibody on the development of EAT. Thirty-six rats were randomly classified into five groups: Sham + PBS (Sh- PBS, *n* = 6), EA + PBS (EA-PBS, *n* = 6), EA + IgG (EA-IgG, *n* = 6), EA + 0.1 μg Tβ4 antibody (EA-0.1 μg Ab, *n* = 6), EA + 1 μg Tβ4 antibody (EA-1 μg Ab, *n* = 6), and EA + 10 μg Tβ4 antibody(EA-10 μg Ab, *n* = 6). The rats in EA-0.1 μg Ab, EA-1 μg Ab, EA-10 μg Ab, or EA-IgG group were intracerebroventricularly injected with 15 μL 0.1 μg, 1 μg, 10 μg Tβ4 neutralizing antibody or isotype IgG, respectively. The rats in Sh-PBS and EA-PBS groups were treated with 15 μL PBS. The rats except those in Sh-PBS group were treated with EA 30 min after intracerebroventricular (icv) injection, for total 8 times. The rats in Sh-PBS group were treated as the same as the rats in EA-PBS group, but without electricity. TFL was examined every day immediately before and after EA, respectively.

**Figure 1:**
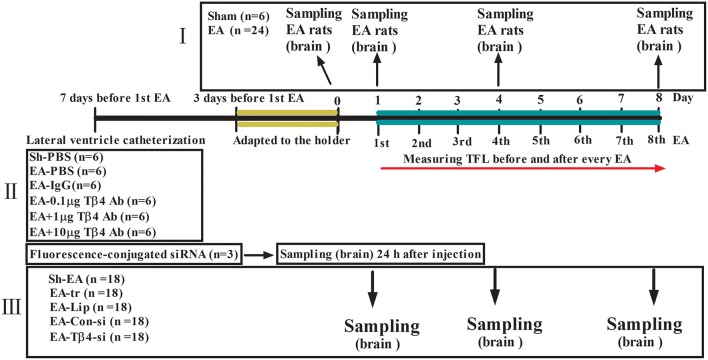
The scheme of experiment. **(I)** The rats in EA group were treated with EA (2/15 Hz, 30 min) once per day for 8 days consecutively. The rats in sham group were treated in the same manner as the rats in EA group, but without electricity. **(II)** The rats in EA-0.1 μg Ab, EA-1 μg Ab, EA-10 μg Ab, or EA-IgG group were intracerebroventricularly injected with 15 μL 0.1 μg, 1 μg, 10 μg Tβ4 neutralizing antibody or isotype IgG, respectively. The rats in Sh-PBS and EA-PBS groups were treated with 15 μL PBS. The rats except those in Sh-PBS group were treated with EA (2/15 Hz, 30 min) 30 min after intracerebroventricular (icv) injection, for total 8 times. The rats in Sh-PBS group were treated as the same as the rats in EA-PBS group, but without electricity. **(III)** The rats in EA-L, EA-C-si, or EA-Tβ4-si group were intracerebroventricularly injected with lipofection, lipofection mixture with control siRNA or Tβ4 siRNA, respectively. The rats except those in Sh-EA group were treated with EA (2/15 Hz, 30 min) at the day after icv injection and thereafter every day, for total 8 times. The rats in Sh-EA group were treated as the same as the rats in EA-tr group, but without electricity. The tail flick latency (TFL) was examined every day immediately before and after EA, respectively. The symbols have the same meanings in Figures 2–5.

To further detect the effect of Tβ4 silencing on the formation of EAT and expression pattern of Tβ4, endorphin (END), encephalin (ENK), dynorphin (DYN), CCK-8, OFQ, MOR, and CCKBR in EA-treated rats, 93 rats were randomly classified into five groups: sham EA (Sh-EA, *n* = 18), EA treatment (EA-tr, *n* = 18), EA treated with lipofection (EA-L, *n* = 18), and EA treated with lipofection mixture with control siRNA (EA-C-si, *n* = 18), or Tβ4 siRNA (EA-Tβ4-si, *n* = 18). The rats in EA-L, EA-C-si, or EA-Tβ4-si group were intracerebroventricularly injected with lipofection (15 μL), lipofection (10 μL) mixture with control siRNA (5 μL) or Tβ4 siRNA (5 μL), respectively. The rats except those in Sh-EA group were treated with EA at the day after icv injection and thereafter every day, for total 8 times. The rats in Sh-EA group were treated as the same as the rats in EA-tr group, but without electricity. TFL was examined every day immediately before and after EA, respectively. Six rats from each group at day 1, 4, and 8, respectively, were euthanized. Additional three rats were used to verify a fluorescence-conjugated siRNA transfection into the brain at 24 h after icv injection (Figure 1).

### Intracerebroventricular Injection

The surgery for icv injection was conducted based on a previous method (Cui et al., 2017a). Briefly, rats were anesthetized with sodium pentobarbital (40 mg/kg, Sigma, USA) and then mounted on a stereotaxic apparatus (RWD, Shenzhen, China). Skin over the skull was incised, and a small hole was micro-drilled on the skull 1.5 mm lateral to and 0.8 mm posterior to the bregma. Then, a small cannula was inserted 4.0 mm below the skull surface and placed in the position. All rats were allowed for recovery for 7 days and for adaption to the cylinder for 3 days before the formal experiment. For icv administration, an injection needle was inserted into the cannula to reach the target site. PBS, Tβ4 antibody (Santa Cruz, CA, USA), lipofection (Santa Cruz, CA, USA) or lipofection mixture with control siRNA (Santa Cruz, CA, USA) / Tβ4 siRNA (Santa Cruz, CA, USA) was administered at a rate of 1 μL/15 min. After injection, the needle was kept in place for 5 min to reduce the backflow of the solution. To verify if siRNA could be transfected into brain areas after icv injection, a fluorescence-conjugated siRNA (Santa Cruz, CA, USA) was used in rats (n = 3).

### Electroacupuncture Application

EA stimulation was conducted at a fixed time of a day (9:00 a.m.), according to the method reported by Cui et al. (2017a). Briefly, each rat was gently placed into a specially designed polyethylene holder, with the hind legs and tail exposed. Before EA, rats were adapted to the holder for 3 days, and the room temperature was controlled at 22 ± 1°C. The skin of the hind legs was sterilized with 75% alcohol. Stainless-steel needles (0.30 mm in diameter, 13 mm in length) were inserted into bilateral Zusanli points (ST36, 4 mm lateral to the anterior tuber point of the tibia, which is marked by a notch, 6–7 mm depth) and Sanyinjiao points (SP6, 3 mm proximal to the medial malleolus at the posterior border of the tibia, 4–5 mm depth). Rats were administered electrical impulses for 30 min with WQ-6F Electronic Acupunctoscope (Beijing Xindonghua Electronic Instrument Co., Ltd., Beijing, China). The stimuli were set as square waves with 2/15 Hz in frequency (dense-and disperse- mode) and 3 mA in amplitude. Throughout the EA, rats were kept in the holder without anesthesia.

### Measurement of Tail Flick Latency

The nociceptive threshold was assessed using the TFL response elicited by radiant heat with the YLS-12A Tail Flick Analgesia Instrument (ZS Dichuang Science and Technology Development Co., Ltd., Beijing, China). Focused light from a projection bulb was applied to the proximal third of the tail and the TFL was measured. The intensity of the thermal stimulus was adjusted to obtain a basal TFL within the range of 4–6 s. A cutoff limit of 15 s was set to avoid tissue damage. Before EA, the basal TFL was determined by averaging three consecutive measures at 5-min intervals. TFL after EA was measured every 10 min during the 30 min of EA application. The change rate of TFL was taken as EA-induced antinociception and was calculated as the formula: TFL (%) = (TFL after EA–basal TFL)/basal TFL × 100%.

### Sample Collection

**Figure 2:**
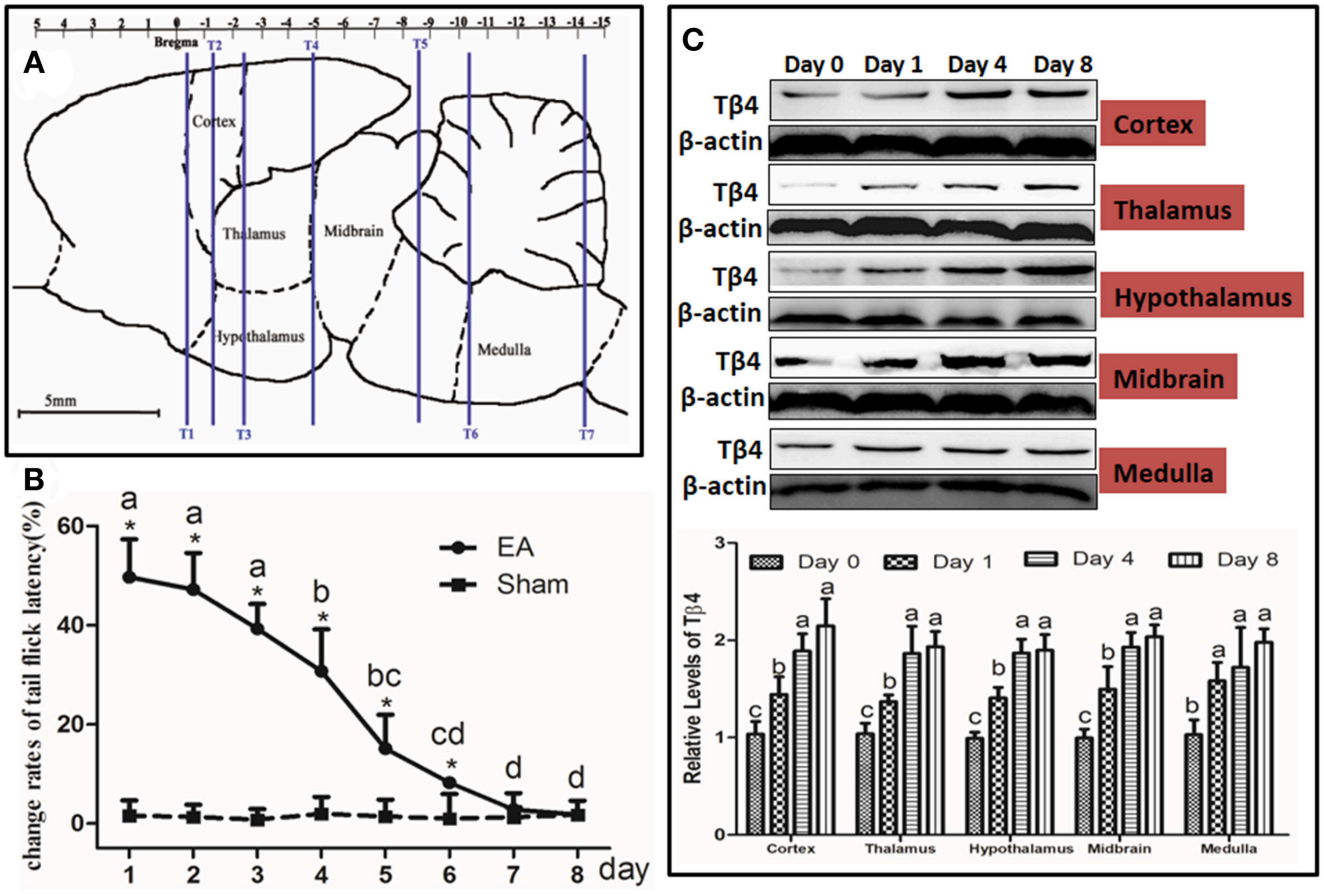
Electroacupuncture (EA)-induced Tβ4 expressions in the brain areas and change rates of tail flick latency. **(A)** Brain areas sampling. The areas to be observed were hypothalamus, thalamus, cortex (sensory region), midbrain and medulla. T1, T2, T3, T4, T5, T6, and T7: marker tubes, show transverse planes at −0.40, −1.30, −2.40, −4.80, −8.60, −10.30, and −14.2 mm rostral to the transverse plane of bregma, respectively. **(B)** Change rates of tail flick latency after repeated EA (Mean ± SD, %, *n* = 6). The significance of differences was calculated by a *t*-test between sham and EA groups or a one-way ANOVA among different time points in EA group followed by Bonferroni's post-test.^*^P < 0.05 compared with sham group. The values with different letters (a–d) within the EA treatment differ. **(C)** The Tβ4 expressions in the brain areas after repeated EA (Mean ± SD, *n* = 6). The values with different letters differ significantly among different time points within the same brain area (*P* < 0.05). One-way ANOVA followed by Bonferroni's post-test.

At day 1, 4, and 8, immediately after TFL measurement, six rats from each group were anesthetized and euthanized by an overdose of sodium pentobarbital and their brains were quickly removed on a DEPC water-treated icebox. According to the atlas of Paxinos and Watson (1986), the hypothalamus, thalamus, cortex (sensory region), midbrain, medulla were taken immediately and stored in liquid nitrogen for western blotting and qPCR detection (Figure 2A).

Three rats were euthanized by an overdose of sodium pentobarbital at 24 h after fluorescence-conjugated siRNA injection, and intracardiac perfused ice-cold 4% paraformaldehyde in PBS. The brain was removed, cryoprotected in 30% sucrose in PBS and embedded in TissuTek. Cryostat sections (10 μm) were collected on slides and observed with an OLYMPUS BX51 microscope with fluorescence light.

### Western Blotting

Brain sample was weighed, grinded in liquid nitrogen, then protein extracted from the grinded brain tissue using the RIPA buffer according to the manufacturer's instruction (Beyotime Biotech, Nantong, China). The protein concentration was measured with Nano Drop Spectrophotometer (Thermo Fisher Scientific, Inc., USA). Equal amounts of protein sample (40 μg) was loaded in 15% SDS polyacrylamide gel and transferred to a PVDF membrane with the Mini-PROTEIN Tetra Cell (Bio-Rad, CA, USA). The membrane was blocked for 2 h at the room temperature in 5% skimmed milk, and was subsequently immunolabeled overnight at 4°C with rabbit anti-endorphin IgG (1:500, ABclonal, Wuhan, China), rabbit anti-enkephalin IgG (1:500, ABclonal, Wuhan, China), rabbit anti-dynorphin IgG (1:500, ABclonal, Wuhan, China), rabbit anti-CCK-8 IgG (1:500, ABclonal, Wuhan, China), rabbit anti-OFQ IgG (1:500, ABclonal, Wuhan, China), rabbit anti-CCKBR IgG (1:500, ABclonal, Wuhan, China), mouse anti-MOR IgG (1:1,000, Novus, CO, USA), mouse anti-Tβ4 IgG (1:300, Santa Cruze, CA, USA), or rabbit anti-beta-actin (1:2,000, Boster biotech, Wuhan, China), respectively. The membrane was washed and treated with horseradish-peroxidase-conjugated anti-rabbit secondary antibody (1:5,000, Boster biotech, Wuhan, China) for 1 h at the room temperature. Visualization of the antigen-antibody complex was conducted with a horseradish peroxidase substrate (Millipore, MA, USA) with the Image Quant LAS 4,000 min CCD camera (GE Healthcare, CHI, USA). The bands were analyzed with Quantity One software (Bio-Rad, CA, USA). Beta-actin was used as the internal control. Values of these substances were represented as the ratio of the optical density of the bands to the density of the related beta-actin band.

### RT-PCR

Total RNA was extracted from the each brain area of each group using Trizol reagent (Invitrogen, CA, USA). Subsequently, cDNA was synthesized from 900 ng of total RNA using a First Strand cDNA Synthesis Kit (TOYOBO, Osaka, Japan). The primer sequences of proopiomelanocortin (POMC), proenkephalin (PENK), prodynorphin (PDYN), CCK, prepronociceptin (PONC), Tβ4, MOR, CCKBR, and GAPDH were shown in Table 1. RT-PCR was performed with Step One Plus™ Real-Time PCR System (Applied Biosystems, CA, USA) using SYBR Green RT-PCR kit (Takara, Dalian, China). The mRNA of POMC, PDYN, PENK, CCK-8, PONC, Tβ4, MOR, and CCKBR relative to GAPDH mRNA were quantified with the 2^−Δ*Ct*^ method, where ΔCt = Ct _targetgene_–Ct _GAPDH_.

**Table 1:** Primer sequences of opioid- and anti-opioid peptide and related receptor genes.

**Name**	**Accession number**	**Primer sequence**
Tβ4	NM_031136	F:5′-GGCTGAGATCGAGAAATT-3′
		R:5′-CTTTTGAAGGCAGAGGAT-3′
PNOC	U48262	F:5′-GTCCGCTGCTCTTTACCA-3′
		R:5′-TGCTTCTGCTCCACCTCAT-3′
CCK	NM_012829	F:5′-TGTCTGTGCGTGGTGATGG-3′
		R:5′-AGGGAGCTTTGCGGACCTG-3′
CCKBR	NM_013165	F:5′-GCCTAAGAACGGTCACCAACG-3′
		R:5′-GACTGTGCCGAAGATGAATGTG-3′
PENK	NM_017139	F:5′-GTGGAGCCAGAAGAAGAGG-3′
		R:5′-CAGCAGGTCGGAGGAGTT-3′
PDYN	NM_019374	F:5′-CGGAGGAGTGGGAGACAT-3′
		R:5′-TGAGACGCTGGTAAGGAGTT-3′
POMC	NM_139326	F:5′-CCTCCTGCTTCAGACCTCCA-3′
		R:5′-GGCTGTTCATCTCCGTTGC-3′
MOR	NM_013071	F:5′-GCTATCGGGCTCCAAAGAA-3′
		R:5′-GCAGAAGTGCCAGGAAACG-3′
GAPDH	NM_017008	F:5′-GTTCAACGGCACAGTCAA-3′
		R:5′-CTCGCTCCTGGAAGATGG-3′

*Tβ4, Thymosin beta 4; POMC, proopiomelanocortin; PENK, proenkephalin; PDYN, prodynorphin; PONC, prepronociceptin; CCK, cholecystokinin; MOR, mu opioid receptor; and CCKBR, CCK B receptor*.

### Statistical Analysis

All data were expressed as mean ± SD. Statistical analyses were performed with SPSS version 18.0 (SPSS Inc., CHI, USA). Independent *T*-test was used to analyze variables, including Tβ4 and its mRNA levels between EA treatment and sham treatment. One-way ANOVA was used to analyze protein and mRNA levels of opioid and anti-opioid peptides and related receptors among the groups with different treatments. Data including TFL changes after repeated EA were analyzed with repeated ANOVA. Bonferroni post-test was used when significant differences were found. The correlation coefficient (Pearson's) was used to examine the correlations. A difference was considered significant if *P* was < 0.05.

## Results

### Repeated EA-Induced Tβ4 Expression and Change Rate of Tail Flick Latency

The TFL was measured every day throughout the experiment (Figure 2B). No change (*P* > 0.05) was found in TFL in sham treatment during this trial. EA-induced TFL change rate was 49.6 ± 7.6% at day 1, then declined (*P* < 0.001) to 30.7 ± 8.5% at day 4 and fell (*P* < 0.001) to 1.8 ± 1.2% at day 8, implying EAT formation. TFL change rates in EA treatment were higher (*P* < 0.05) than those in sham treatment at day 1 to 6.

The dynamic expression of Tβ4 induced by repeated EA was determined in cortex, thalamus, hypothalamus, midbrain and medulla at day 0, 1, 4, and 8. EA increased (*P* < 0.05) the expression of Tβ4 in the measured areas at day 1–8 (Figure 2C). Statistical analysis showed that TFL change rates had a negative correlation with Tβ4 levels in cortex (*r* = −0.774, *P* < 0.001), in thalamus (*r* = −0.689, *P* = 0.002), in hypothalamus (*r* = −0.705, *P* = 0.001), in midbrain (*r* = −0.709, *P* = 0.001) and in medulla (*r* = −0.612, *P* = 0.007).

### The Effect of Intracerebroventricularly Injection of Tβ4 Antibody on Repeated EA-Induced Tail Flick Latency

**Figure 3:**
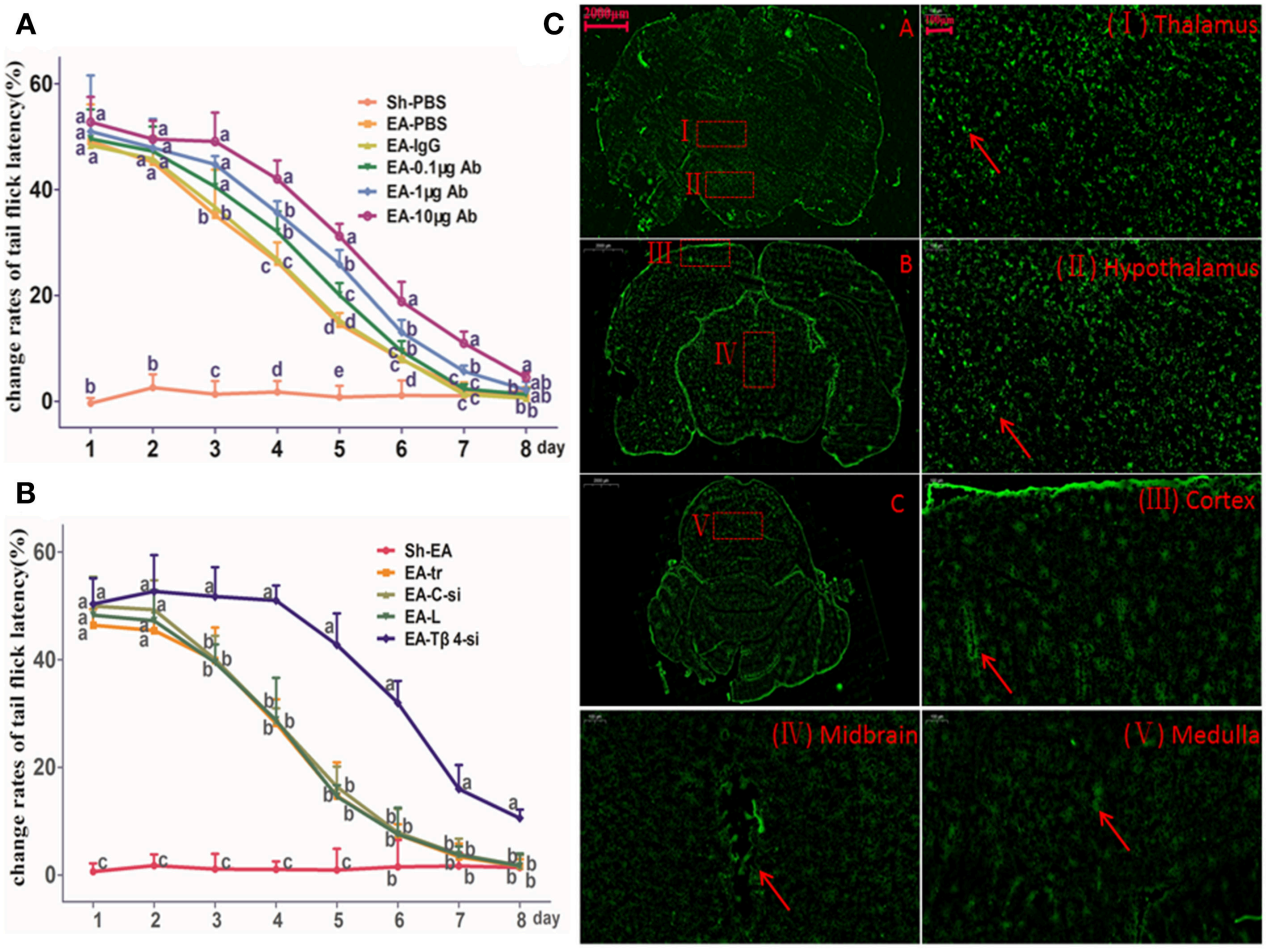
The effect of intracerebroventricularly (icv) injection of Tβ4 antibody or siRNA on electroacupuncture (EA)-induced tail flick latency (TFL). **(A)** The effect of icv injection of Tβ4 antibody on change rates of TFL (mean ± SD, %, *n* = 6). The values with different letters differ significantly in different groups at the same day (*P* < 0.05). One-way ANOVA followed by Bonferroni's post-test. **(B)** The effect of icv injection of Tβ4 siRNA on change rates of TFL (mean ± SD, %, *n* = 6). The values with different letters differ significantly in different groups at the same day (*P* < 0.05). One-way ANOVA followed by Bonferroni's post-test. **(C)** The fluorescence in cortex, thalamus, hypothalamus, mid-brain and medulla. Arrows point to the high fluorescence intensity. Scale bars represent 2,000 μm in picture **(A–C)**, and 100 μm in others.

TFL change rates in Sh-EA and EA-PBS groups showed the same change pattern as those in Figure 2B. TFL change rate of EA-treated rats was decreased in a Tβ4-antibody-dose- and time-dependent manner. EA-treated rats with icv injection of Tβ4 antibody showed higher (*P* < 0.05) TFL change rates than rats with PBS or isotype IgG at day 3–7. TFL change rates in 10 μg antibody group were higher (*P* < 0.05) than that in 1 μg or 0.1 μg antibody group at day 4–7 (Figure 3A).

### The Effect of Tβ4 siRNA on Repeated EA-Induced Tail Flick Latency

To verify siRNA was able to transfect, the fluorescence-conjugated control siRNA was intracerebroventricularly injected, the fluorescence in hypothalamus, thalamus, cortex, midbrain, and medulla was observed at 24 h after injection (Figure 3B).

TFL change rates in Sh-EA and EA-tr groups showed the same change pattern as those in Figure 2B, and the development of EAT at day 8. TFL change rates in EA-tr, EA-L, and EA-C-si groups were higher (*P* < 0.05) than those in Sh-EA group at day 1–5, but lower (*P* < 0.05) than those in EA-Tβ4-si group at day 3–8. No change was observed in TFLs among EA-tr, EA-L, and EA-C-si groups (Figure 3C).

### The Effect of Tβ4 siRNA on Protein Expressions of Opioid- and Anti-opioid Peptides and Related Receptors in the Brain Areas of Rats With Repeated EA

The protein expressions of END, ENK, DYN, CCK-8, OFQ, Tβ4, MOR, and CCKBR were observed in hypothalamus, thalamus, cortex, midbrain and medulla at day 1–8. No difference (*P* > 0.05) was observed in the expressions of these eight substances among EA-tr, EA-L, and EA-C-si groups in the measured areas during the experiment.

**Figure 4:**
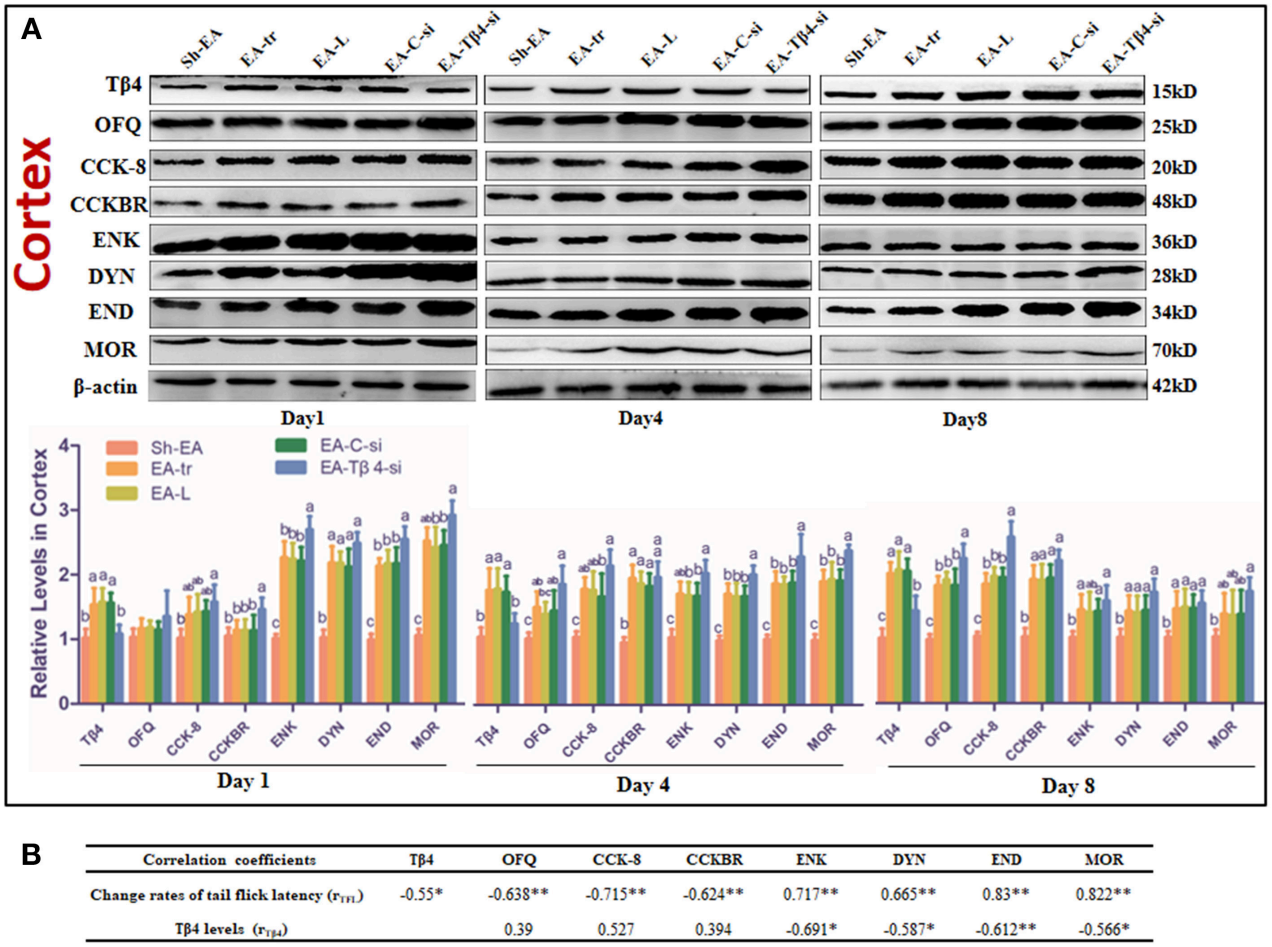
**(A)** The effect of Tβ4 siRNA on the levels of opioid and anti-opioid peptides and their receptors in the cortex of rats with repeated EA on day 1, 4, and 8 (mean ± SD, *n* = 6). The expressions of thymosin beta 4 (Tβ4), orphanin FQ (OFQ), cholecystokinin octapeptide (CCK-8), CCK B receptor (CCKBR), endorphin (END), encephalin (ENK), dynorphin (DYN), and mu opioid receptor (MOR) were observed. The values with different letters (a-c) differ significantly in different groups at the same day (*P* < 0.05). One-way ANOVA followed by Bonferroni's post-test. **(B)** The correlation coefficients between the levels of opioid or anti-opioid peptides or their receptors and the change rates of tail flick latency (TFL) or Tβ4 levels. ^*^P < 0.05, ^*^^*^P < 0.01. The correlation coefficient was analyzed with Pearson's correlation coefficient.

The protein levels of those substances in cortex were shown in Figure 4A. Compared with sham treatment, EA treatment induced an increase in OFQ, CCK-8, and CCKBR levels (day 4 and 8), and Tβ4, ENK, DYN, and END levels (day 1–8), and MOR level (day 1 and 4). EA-Tβ4 siRNA-treated rats exhibited lower Tβ4 level (day 1–8), but higher levels of CCKBR (day 4), DYN (day 1), ENK, END, and MOR (day 1 and 4) than EA-treated rats. TFL change rates were negatively (*P* < 0.05) correlated with Tβ4, OFQ, CCK-8, or CCKBR levels, but positively (*P* < 0.05) correlated with ENK, DYN, END, or MOR levels. Tβ4 levels were negatively correlated with ENK, DYN, END, or MOR levels (Figure 4B).

**Figure 5:**
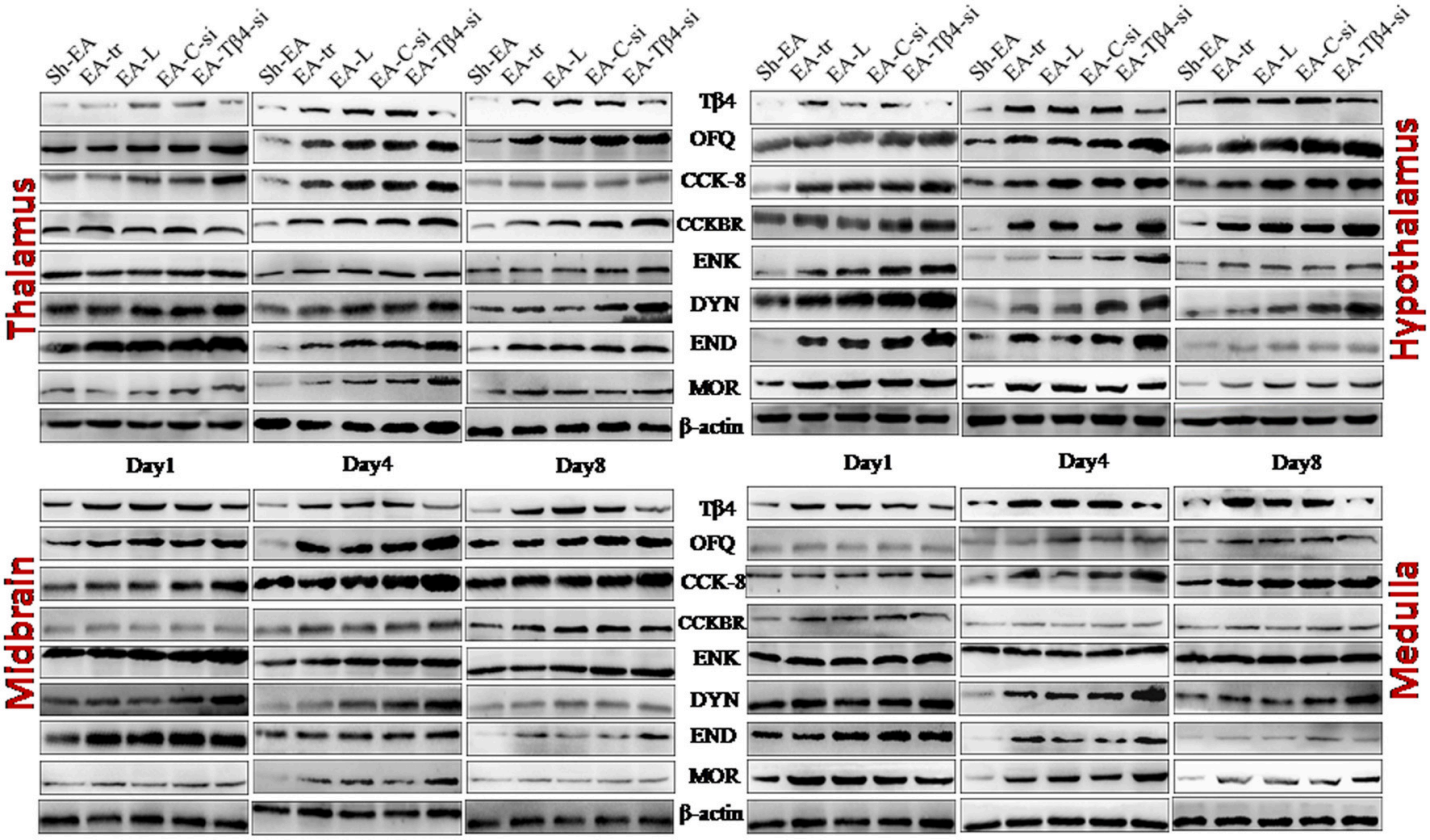
The represented bands of Tβ4, OFQ, CCK-8, CCKBR, ENK, END, DYN, and MOR detected with western blotting in the thalamus, hypothalamus, midbrain and medulla of rats on day 1, 4, and 8.

In thalamus, compared with Sh-EA treated rats, EA-treated rats showed an increase in Tβ4 and OFQ (day 1–8), and CCK-8 and CCKBR (day 4 and 8), and ENK, DYN, END, and MOR (day 1 and 4). Compared with EA-treated rats, **EA**-Tβ4 siRNA-treated rats showed a decrease in Tβ4 (day 1–8), but an increase in OFQ and ENK (day 4 and 8), DYN and MOR (day 1–8), and END (day 4). TFL change rates were negatively (*P* < 0.05) correlated with Tβ4, OFQ, CCK-8, or CCKBR levels, but positively (*P* < 0.05) correlated with ENK, DYN, END, or MOR levels. Tβ4 levels were positively correlated with OFQ levels, but negatively correlated with ENK, DYN, END, END, or MOR levels (Table 2).

**Table 2:** The effect of Tβ4 siRNA on protein levels of opioid- and anti-opioid peptides and related receptors.

**Areas**	**Day 1**				**Day 4**				**Day 8**							**Correlation coefficients**	
	**Sh-EA**	**EA-tr**	**EA-L-si**	**EA-C-si**	**EA-Tβ4-si**	**Sh-EA**	**EA-tr**	**EA-L-si**	**EA-C-si**	**EA-Tβ4-si**	**Sh-EA**	**EA-tr**	**EA-L-si**	**EA-C-si**	**EA-Tβ4-si**	**r_**(TFL)**_**	**r_**(*Tβ*4)**_**
**THALAMUS**																	
Tβ4	1.01 ± 0.06^b^	1.5 ± 0.39^a^	1.52 ± 0.29^a^	1.53 ± 0.21^a^	1.24 ± 0.08^b^	1.04 ± 0.12^b^	1.83 ± 0.23^a^	1.81 ± 0.24^a^	1.78 ± 0.22^a^	1.25 ± 0.19^b^	1.04 ± 0.11^c^	1.94 ± 0.16^a^	1.97 ± 0.13^a^	1.94 ± 0.15^a^	1.51 ± 0.23^b^	−0.637^**^	
OFQ	0.99 ± 0.1^b^	1.58 ± 0.11^a^	1.52 ± 0.17^a^	1.56 ± 0.18^a^	1.6 ± 0.29^a^	1 ± 0.12^c^	1.52 ± 0.12^b^	1.5 ± 0.2^b^	1.52 ± 0.29^b^	1.91 ± 0.19^a^	1.02 ± 0.11^c^	1.87 ± 0.16^a^	1.78 ± 0.32^a^	1.83 ± 0.14^a^	2.23 ± 0.19^b^	−0.677^**^	0.563^*^
CCK-8	1.05 ± 0.11	1.04 ± 0.17	1.06 ± 0.14	1.03 ± 0.18	1.23 ± 0.22	1.01 ± 0.12^b^	1.42 ± 0.21^a^	1.43 ± 0.23^a^	1.48 ± 0.18^a^	1.77 ± 0.22^a^	1.05 ± 0.13^b^	1.63 ± 0.14^a^	1.63 ± 0.09^a^	1.62 ± 0.15^a^	1.92 ± 0.28^a^	−0.527^*^	0.369
CCKBR	0.99 ± 0.09	1.1 ± 0.18	1.12 ± 0.13	1.19 ± 0.08	1.2 ± 0.25	1.01 ± 0.09^b^	1.8 ± 0.12^a^	1.84 ± 0.18^a^	1.72 ± 0.25^a^	1.74 ± 0.53^a^	1 ± 0.09^b^	1.84 ± 0.18^a^	1.88 ± 0.16^a^	1.86 ± 0.19^a^	2.02 ± 0.2^a^	−0.515^*^	0.086
ENK	1.01 ± 0.11^b^	2.01 ± 0.13^a^	1.97 ± 0.13^a^	1.99 ± 0.19^a^	2.25 ± 0.22^a^	1.01 ± 0.13^c^	1.53 ± 0.16^b^	1.52 ± 0.14^b^	1.48 ± 0.18^b^	2 ± 0.1^a^	1.01 ± 0.12^b^	1.2 ± 0.12^b^	1.18 ± 0.08^b^	1.2 ± 0.14^b^	1.57 ± 0.19^a^	0.837^**^	−0.690^*^
DYN	1 ± 0.11^c^	2.28 ± 0.23^b^	2.11 ± 0.15^b^	2.1 ± 0.16^b^	2.62 ± 0.24^a^	1.01 ± 0.14^c^	1.66 ± 0.24^b^	1.63 ± 0.19^b^	1.66 ± 0.21^b^	2.17 ± 0.19^a^	1.04 ± 0.11^b^	1.23 ± 0.14^b^	1.22 ± 0.12^b^	1.26 ± 0.13^b^	1.68 ± 0.21^a^	0.785^**^	−0.530^*^
END	1.03 ± 0.12^b^	2.55 ± 0.28^a^	2.58 ± 0.21^a^	2.55 ± 0.39^a^	2.86 ± 0.26^a^	1.07 ± 0.12^c^	1.72 ± 0.21^b^	1.68 ± 0.35^b^	1.68 ± 0.2^b^	2.21 ± 0.24^a^	1.02 ± 0.17	1.08 ± 0.27	0.99 ± 0.15	1.01 ± 0.23	1.26 ± 0.28	0.874^**^	−0.713^**^
MOR	1.04 ± 0.11^c^	2.34 ± 0.22^b^	2.31 ± 0.2^b^	2.28 ± 0.22^b^	2.82 ± 0.16^a^	1 ± 0.11^c^	1.76 ± 0.16^b^	1.75 ± 0.15^b^	1.77 ± 0.19^b^	2.25 ± 0.17^a^	1 ± 0.04^b^	1.28 ± 0.13^b^	1.24 ± 0.17^b^	1.21 ± 0.17^b^	1.9 ± 0.26^a^	0.668^**^	−0.537^*^
**HYPOTHALAMUS**																	
Tβ4	1.04 ± 0.11^b^	1.47 ± 0.21^a^	1.48 ± 0.22^a^	1.49 ± 0.21^a^	1.13 ± 0.07^b^	1.02 ± 0.14^c^	1.92 ± 0.17^a^	1.93 ± 0.28^a^	1.92 ± 0.35^a^	1.45 ± 0.09^b^	1.04 ± 0.07^b^	2.18 ± 0.3^a^	2.16 ± 0.24^a^	2.12 ± 0.25^a^	1.87 ± 0.06^a^	−0.868^**^	
OFQ	1 ± 0.13^b^	1.3 ± 0.08^a^	1.33 ± 0.09^a^	1.3 ± 0.11^a^	1.49 ± 0.17^a^	1.03 ± 0.14^b^	1.68 ± 0.3^a^	1.69 ± 0.2^a^	1.51 ± 0.13^a^	1.87 ± 0.27^a^	0.98 ± 0.08^c^	1.86 ± 0.12^b^	1.87 ± 0.09^b^	1.85 ± 0.11^b^	2.19 ± 0.28^a^	−0.65^**^	0.788^**^
CCK-8	1.03 ± 0.08^b^	1.36 ± 0.11^ab^	1.33 ± 0.28^ab^	1.33 ± 0.26^ab^	1.55 ± 0.17^a^	0.99 ± 0.05^b^	1.87 ± 0.22^a^	1.77 ± 0.41^a^	1.82 ± 0.16^a^	2.12 ± 0.16^a^	0.98 ± 0.1^c^	1.73 ± 0.38^b^	1.71 ± 0.21^b^	1.68 ± 0.25^b^	2.28 ± 0.26^a^	−0.493^*^	0.746^**^
CCKBR	0.98 ± 0.06^c^	1.42 ± 0.23^b^	1.44 ± 0.3^b^	1.4 ± 0.19^ab^	1.8 ± 0.18^a^	1.01 ± 0.12^a^	2 ± 0.24^a^	2.06 ± 0.31^a^	1.97 ± 0.32^a^	2.03 ± 0.4^a^	1.1 ± 0.18^b^	1.97 ± 0.4^a^	1.95 ± 0.31^a^	2.03 ± 0.23^a^	2.09 ± 0.21^a^	−0.263	0.321
ENK	1.03 ± 0.09^b^	2.64 ± 0.33^a^	2.76 ± 0.27^a^	2.77 ± 0.21^a^	3.12 ± 0.17^a^	1.03 ± 0.11^c^	1.6 ± 0.13^b^	1.57 ± 0.32^b^	1.57 ± 0.32^b^	2.01 ± 0.17^a^	1 ± 0.04^b^	1.29 ± 0.28^b^	1.33 ± 0.12^b^	1.32 ± 0.13^b^	1.65 ± 0.21^a^	0.647^**^	−0.879^**^
DYN	1.01 ± 0.08^b^	2.31 ± 0.26^a^	2.34 ± 0.29^a^	2.42 ± 0.39^a^	2.63 ± 0.27^a^	1.01 ± 0.04^c^	1.49 ± 0.2^b^	1.47 ± 0.32^b^	1.45 ± 0.23^b^	1.99 ± 0.11^a^	1.02 ± 0.07^b^	1.13 ± 0.18^b^	1.14 ± 0.33^b^	1.05 ± 0.28^b^	1.7 ± 0.17^a^	0.638^**^	−0.853^**^
END	1.03 ± 0.09^b^	2.74 ± 0.28^a^	2.74 ± 0.23^a^	2.79 ± 0.17^a^	3.01 ± 0.34^a^	1.02 ± 0.07^c^	1.51 ± 0.27^b^	1.57 ± 0.21^b^	1.58 ± 0.26^b^	2.14 ± 0.11^a^	1 ± 0.04^b^	1.31 ± 0.22^ab^	1.29 ± 0.28^ab^	1.27 ± 0.23^ab^	1.56 ± 0.19^a^	0.757^**^	−0.912^**^
MOR	1.04 ± 0.09^b^	2.73 ± 0.3^a^	2.74 ± 0.3^a^	2.83 ± 0.26^a^	3.2 ± 0.32^a^	1 ± 0.11^b^	1.72 ± 0.16^a^	1.72 ± 0.17^a^	1.76 ± 0.17^a^	2.27 ± 0.35^a^	1 ± 0.04^c^	1.23 ± 0.19^b^	1.19 ± 0.23^b^	1.24 ± 0.19^b^	1.9 ± 0.23^a^	0.622^**^	−0.836^**^
**MIDBRAIN**																	
Tβ4	1.01 ± 0.11^b^	1.48 ± 0.25^a^	1.51 ± 0.27^a^	1.48 ± 0.22^a^	1.06 ± 0.16^b^	0.98 ± 0.11^c^	1.95 ± 0.17^a^	1.98 ± 0.35^a^	1.95 ± 0.22^a^	1.44 ± 0.07^b^	0.98 ± 0.11^c^	1.99 ± 0.11^a^	2.07 ± 0.3^a^	1.97 ± 0.11^a^	1.51 ± 0.16^b^	−0.505^*^	
OFQ	1.02 ± 0.07^c^	1.41 ± 0.18^b^	1.46 ± 0.18^b^	1.45 ± 0.17^b^	1.86 ± 0.32^a^	1.01 ± 0.11^c^	1.53 ± 0.21^b^	1.54 ± 0.27^b^	1.59 ± 0.27^b^	2.06 ± 0.24^a^	1.03 ± 0.06^b^	1.72 ± 0.24^a^	1.78 ± 0.3^a^	1.77 ± 0.19^a^	2.04 ± 0.27^a^	−0.154	0.065
CCK-8	1.04 ± 0.07^c^	1.52 ± 0.24^b^	1.53 ± 0.2^b^	1.57 ± 0.25^b^	1.98 ± 0.14^a^	1 ± 0.11^c^	1.79 ± 0.17^b^	1.79 ± 0.2^b^	1.79 ± 0.25^b^	2.29 ± 0.33^a^	1.03 ± 0.07^b^	2.08 ± 0.25^a^	2.06 ± 0.27^a^	2.07 ± 0.26^a^	2.33 ± 0.18^a^	−0.275	0.518^*^
CCKBR	1.04 ± 0.07^b^	1.47 ± 0.29^a^	1.42 ± 0.25^ab^	1.45 ± 0.27^a^	1.53 ± 0.21^a^	1.06 ± 0.12^b^	1.42 ± 0.31^a^	1.42 ± 0.26^a^	1.47 ± 0.24^a^	1.83 ± 0.2^a^	1.01 ± 0.08^b^	1.9 ± 0.3^a^	1.93 ± 0.26^a^	1.92 ± 0.58^a^	1.97 ± 0.49^a^	−0.375	0.451
ENK	1.02 ± 0.13^b^	2.05 ± 0.28^a^	2.1 ± 0.35^a^	2.07 ± 0.35^a^	2.41 ± 0.3^a^	0.99 ± 0.1^c^	1.47 ± 0.15^b^	1.45 ± 0.26^b^	1.47 ± 0.41^b^	1.99 ± 0.14^a^	0.94 ± 0.12^c^	1.35 ± 0.26^b^	1.38 ± 0.29^b^	1.38 ± 0.21^b^	1.88 ± 0.15^a^	0.478^*^	−0.811^**^
DYN	1.05 ± 0.15^b^	2.21 ± 0.2^a^	2.22 ± 0.4^a^	2.22 ± 0.28^a^	2.64 ± 0.2^a^	1 ± 0.09^c^	1.85 ± 0.23^b^	1.92 ± 0.24^b^	1.9 ± 0.22^b^	2.34 ± 0.25^a^	1.04 ± 0.12^b^	1.44 ± 0.27^a^	1.5 ± 0.27^a^	1.44 ± 0.36^a^	1.82 ± 0.16^a^	0.818^**^	−0.674^**^
END	1.05 ± 0.14^b^	2.13 ± 0.29^a^	2.11 ± 0.34^a^	2.18 ± 0.33^a^	2.51 ± 0.31^a^	1.02 ± 0.14^c^	1.85 ± 0.19^b^	1.87 ± 0.21^b^	1.89 ± 0.34^b^	2.34 ± 0.27^a^	0.96 ± 0.11^c^	1.41 ± 0.24^b^	1.48 ± 0.25^ab^	1.42 ± 0.32^b^	1.85 ± 0.16^a^	0.752^**^	−0.736^**^
MOR	1.02 ± 0.11^b^	2.65 ± 0.19^a^	2.69 ± 0.22^a^	2.66 ± 0.16^a^	2.97 ± 0.27^a^	1.03 ± 0.07^c^	2.04 ± 0.35^b^	2 ± 0.29^b^	2 ± 0.38^b^	2.66 ± 0.23^a^	1.02 ± 0.09^b^	1.39 ± 0.35^b^	1.42 ± 0.26^b^	1.41 ± 0.07^b^	1.89 ± 0.29^a^	0.857^**^	−0.607^**^
**MEDULLA**																	
Tβ4	1.01 ± 0.12^b^	1.3 ± 0.17^a^	1.3 ± 0.2^a^	1.29 ± 0.28^a^	0.86 ± 0.16^b^	1.03 ± 0.14^b^	1.5 ± 0.21^a^	1.44 ± 0.2^a^	1.49 ± 0.28^a^	1.18 ± 0.22^b^	1.04 ± 0.14^c^	1.68 ± 0.33^a^	1.62 ± 0.21^ab^	1.67 ± 0.21^a^	1.25 ± 0.22^b^	−0.476^*^	
OFQ	0.99 ± 0.09^b^	1.49 ± 0.22^a^	1.48 ± 0.24^a^	1.46 ± 0.26^a^	1.47 ± 0.25^a^	1.05 ± 0.12^b^	1.68 ± 0.33^a^	1.7 ± 0.22^a^	1.71 ± 0.23^a^	1.99 ± 0.18^a^	1.02 ± 0.09^b^	1.98 ± 0.23^a^	1.94 ± 0.23^a^	1.92 ± 0.29	2.29 ± 0.25^a^	−0.625^**^	0.565^*^
CCK-8	1.01 ± 0.09^b^	1.45 ± 0.2^a^	1.42 ± 0.22^a^	1.42 ± 0.22^a^	1.77 ± 0.26^a^	1.03 ± 0.13^c^	1.68 ± 0.23^b^	1.68 ± 0.13^b^	1.71 ± 0.17^b^	2.04 ± 0.19^a^	1.05 ± 0.07^b^	1.68 ± 0.23^a^	1.7 ± 0.26^a^	1.7 ± 0.25^a^	2.05 ± 0.39^a^	−0.249	0.215
CCKBR	0.99 ± 0.13^b^	1.52 ± 0.15^a^	1.56 ± 0.27^a^	1.53 ± 0.22^a^	1.58 ± 0.21^a^	1.05 ± 0.09^b^	1.35 ± 0.33^b^	1.35 ± 0.21^b^	1.37 ± 0.23^b^	1.81 ± 0.11^a^	1.03 ± 0.12^b^	1.61 ± 0.43^a^	1.68 ± 0.26^a^	1.7 ± 0.3^a^	1.92 ± 0.23^a^	−0.532^*^	0.395
ENK	1.01 ± 0.1^b^	2.08 ± 0.2^a^	2.12 ± 0.3^a^	2.11 ± 0.23^a^	2.3 ± 0.43^a^	1.04 ± 0.14^c^	1.72 ± 0.2^b^	1.74 ± 0.23^b^	1.77 ± 0.17^b^	2.4 ± 0.26^a^	1.02 ± 0.11^b^	1.47 ± 0.26^a^	1.44 ± 0.3^a^	1.52 ± 0.19^a^	1.74 ± 0.25^a^	0.698^**^	−0.178
DYN	1.02 ± 0.07^b^	2.18 ± 0.28^a^	2.14 ± 0.3^a^	2.2 ± 0.25^a^	2.53 ± 0.34^a^	1.05 ± 0.11^b^	1.68 ± 0.33^a^	1.7 ± 0.23^a^	1.7 ± 0.28^a^	2.13 ± 0.37^a^	1.03 ± 0.07^c^	1.25 ± 0.3^b^	1.28 ± 0.26^b^	1.27 ± 0.22^b^	1.75 ± 0.26^a^	0.586^*^	−0.497^*^
END	1.04 ± 0.12^c^	2.02 ± 0.26^b^	2.09 ± 0.31^b^	2.11 ± 0.28^b^	2.68 ± 0.28^a^	1.03 ± 0.1^c^	1.69 ± 0.26^b^	1.71 ± 0.21^b^	1.7 ± 0.19^b^	2.09 ± 0.15^a^	1 ± 0.11	1.22 ± 0.32	1.24 ± 0.2	1.24 ± 0.18	1.44 ± 0.37	0.79^**^	−0.565^*^
MOR	1.03 ± 0.09^b^	2.24 ± 0.38^a^	2.24 ± 0.26^a^	2.21 ± 0.21^a^	2.4 ± 0.3^a^	1 ± 0.11^c^	1.84 ± 0.23^b^	1.84 ± 0.16^b^	1.85 ± 0.45^b^	2.35 ± 0.15^a^	1.02 ± 0.08^b^	1.22 ± 0.24^b^	1.25 ± 0.3^b^	1.27 ± 0.26^ab^	1.7 ± 0.29^a^	0.773^**^	−0.482^*^

*The measured brain areas included hypothalamus, thalamus, midbrain and medulla. The protein levels of Tβ4, thymosin beta 4; OFQ orphanin; CCK-8, cholecystokinin octapeptide; CCKBR, CCK B receptor; END, endorphin; ENK, encephalin; DYN, dynorphin; MOR, mu opioid receptor were measured with western blotting. The rats in EA-L, EA-C-si, or EA-Tβ4-si group were icv injected with lipofection, lipofection mixture with Control siRNA or Tβ4 siRNA, respectively. The rats except those in Sh-EA group were treated with EA (2/15 Hz, 30 min) at the day after icv injection and thereafter every day, for total 8 times. The rats in Sh-EA group were treated as the same as the rats in EA-tr group, but without electricity. The symbols have the same meanings in Table 3. The values with different letters (a-c) differ significantly in different groups at the same day (P < 0.05). One-way ANOVA followed by Bonferroni's post-test*.

* means the levels of opioid or anti-opioid peptides or their receptors correlate with the change rates of tail flick latency (TFL) or Tβ4 levels at the 0.05 level, and

** *means at the 0.01 level. The correlation coefficient was analyzed with Pearson's correlation coefficient*.

In hypothalamus, rats in EA-tr group showed an increase in CCK-8 (day 4 and 8), and Tβ4, OFQ, CCKBR, and MOR (day 1–8), and ENK, DYN, and END (day 1 and 4) compared with rats in Sh-EA group. **EA**-Tβ4 siRNA treatment caused a decrease (*P* < 0.05) in Tβ4 (day 1 and 4), but an increase in OFQ, CCK-8, and MOR (day 8), in ENK and DYN (day 4 and 8), and END (day 4) and CCKBR (day 1) Compared with EA treatment. TFL change rates were negatively (*P* < 0.05) correlated with Tβ4, OFQ, or CCK-8 levels, but positively (*P* < 0.05) correlated with ENK, DYN, END, or MOR levels. Tβ4 levels were positively correlated with OFQ or CCK-8 levels, but negatively correlated with ENK, DYN, END, or MOR levels (Table 2).

In midbrain, EA treatment caused increased Tβ4, OFQ, CCK-8, CCKBR, ENK, DYN, and END (day 1–8) and MOR levels (day 1 and 4) compared with sham treatment. EA-Tβ4 siRNA treatment induced a decrease in Tβ4 (day 1–8), but an increase in OFQ and CCK-8 (day 1 and 4), ENK, END, and MOR (day 4 and 8), and DYN (day 4) compared with EA treatment. TFL change rates were negatively (*P* < 0.05) correlated with Tβ4 levels, but positively (*P* < 0.05) correlated with ENK, DYN, END, or MOR levels. Tβ4 levels were positively correlated with CCK-8 levels, but negatively correlated with ENK, DYN, END, or MOR levels (Table 2).

In medulla, Tβ4, OFQ, CCK-8, CCKBR, ENK, and DYN levels (day 1–8) and END and MOR levels (day 1 and 4) in EA-tr group were enhanced compared with those in sham group. Rats in EA-Tβ4-si group exhibited a decrease in Tβ4 (day 1–8), but an increase in CCK-8, CCKBR, and ENK (day 4), DYN (day 8), END (day 1 and 4), and MOR (day 4 and 8) compared with rats in EA-tr group. TFL change rates were negatively (*P* < 0.05) correlated with Tβ4, OFQ or CCKBR levels, but positively (*P* < 0.05) correlated with ENK, DYN, END, or MOR levels. Tβ4 levels were positively correlated with OFQ levels, but negatively correlated with ENK, DYN, END, or MOR levels (Table 2).

### The Effect of Tβ4 siRNA on Gene Expressions of Opioid- and Anti-opioid Peptides and Related Receptors in the Brain Areas of Rats With Repeated EA

PNOC, PENK, POMC, and PDYN are the precursors of OFQ, ENK, END, and DYN, respectively. The mRNA levels of POMC, PENK, PDYN, CCK, Tβ4, MOR, and CCKBR are measured in hypothalamus, thalamus, cortex, midbrain, and medulla at day 1–8 (Table 3). No difference (*P* > 0.05) was observed in the expression of these eight genes among EA-tr, EA-L, and EA-C-si groups in the measured areas during the experiment.

**Table 3:** The effect of Tβ4 siRNA on mRNAs of opioid- and anti-opioid peptides and related receptors.

**Areas**	**Day 1**					**Day 4**					**Day 8**				
	**Sh-EA**	**EA-tr**	**EA-L-si**	**EA-C-si**	**EA-Tβ4-si**	**Sh-EA**	**EA-tr**	**EA-L-si**	**EA-C-si**	**EA-Tβ4-si**	**Sh-EA**	**EA-tr**	**EA-L-si**	**EA-C-si**	**EA-Tβ4-si**
**CORTEX**															
Tβ4	1.03 ± 0.12^b^	1.56 ± 0.19^a^	1.53 ± 0.29^a^	1.58 ± 0.13^a^	1.06 ± 0.16^b^	1.04 ± 0.11^c^	2.01 ± 0.12^a^	1.93 ± 0.11^a^	1.95 ± 0.09^a^	1.51 ± 0.22^b^	1.01 ± 0.06^c^	2.17 ± 0.24^a^	2.18 ± 0.19^a^	2.19 ± 0.16^a^	1.74 ± 0.07^b^
PNOC	1.02 ± 0.11^b^	1.44 ± 0.35^ab^	1.23 ± 0.11^ab^	1.5 ± 0.35^ab^	1.8 ± 0.5^a^	1 ± 0.12^c^	1.89 ± 0.1^ab^	1.86 ± 0.11^ab^	1.84 ± 0.1^b^	2.05 ± 0.12^a^	1.05 ± 0.12^b^	1.88 ± 0.36^a^	1.72 ± 0.38^a^	1.82 ± 0.35^a^	1.86 ± 0.3^a^
CCK	1 ± 0.11^b^	1.39 ± 0.14^a^	1.37 ± 0.16^a^	1.31 ± 0.24^ab^	1.58 ± 0.22^a^	0.99 ± 0.13^b^	1.73 ± 0.38^a^	1.71 ± 0.21^a^	1.68 ± 0.25^a^	1.97 ± 0.16^a^	1.05 ± 0.11^b^	1.81 ± 0.37^a^	1.8 ± 0.42^a^	1.73 ± 0.38^a^	1.91 ± 0.36^a^
CCKBR	1.04 ± 0.07^b^	1.36 ± 0.17^ab^	1.31 ± 0.16^ab^	1.33 ± 0.24^ab^	1.39 ± 0.27^a^	1.01 ± 0.08	1.41 ± 0.3	1.43 ± 0.19	1.43 ± 0.23	1.24 ± 0.39	0.99 ± 0.09^b^	1.68 ± 0.38^a^	1.79 ± 0.37^a^	1.65 ± 0.39^a^	1.57 ± 0.36^ab^
PENK	1.01 ± 0.12^b^	1.33 ± 0.22^b^	1.34 ± 0.27^ab^	1.36 ± 0.25^ab^	1.74 ± 0.25^a^	0.94 ± 0.12^c^	1.61 ± 0.2^b^	1.7 ± 0.17^b^	1.68 ± 0.23^b^	2.08 ± 0.2^a^	1.04 ± 0.14	1.03 ± 0.22	0.95 ± 0.32	1.01 ± 0.36	1.03 ± 0.36
PDYN	1.03 ± 0.07^b^	1.26 ± 0.22^b^	1.31 ± 0.31^b^	1.31 ± 0.12^b^	1.73 ± 0.31^a^	1.05 ± 0.11^c^	1.61 ± 0.24^b^	1.63 ± 0.14^b^	1.67 ± 0.22^b^	2.01 ± 0.14^a^	1.01 ± 0.14	1.38 ± 0.28	1.35 ± 0.33	1.3 ± 0.31	1.39 ± 0.18
POMC	1.02 ± 0.14	1.08 ± 0.27	0.99 ± 0.15	1.01 ± 0.23	1.26 ± 0.28	1.07 ± 0.12^b^	1.87 ± 0.24^a^	1.83 ± 0.16^a^	1.9 ± 0.33^a^	2.17 ± 0.15^a^	1.02 ± 0.17^b^	1.48 ± 0.27^a^	1.45 ± 0.26^a^	1.34 ± 0.23^ab^	1.69 ± 0.24^a^
MOR	1 ± 0.1^c^	1.28 ± 0.13^ab^	1.24 ± 0.17^bc^	1.21 ± 0.17^bc^	1.54 ± 0.21^a^	1.01 ± 0.12^c^	1.85 ± 0.08^b^	1.82 ± 0.22^b^	1.88 ± 0.15^b^	2.33 ± 0.16^a^	1 ± 0.11^b^	1.63 ± 0.22^a^	1.67 ± 0.13^a^	1.64 ± 0.21^a^	1.97 ± 0.27^a^
**THALAMUS**															
Tβ4	1.01 ± 0.11^c^	1.42 ± 0.18^a^	1.39 ± 0.24^a^	1.37 ± 0.19^ab^	1.08 ± 0.08^bc^	1.01 ± 0.14^c^	1.84 ± 0.25^a^	1.9 ± 0.33^a^	1.84 ± 0.12^a^	1.34 ± 0.23^b^	1.04 ± 0.12^b^	1.85 ± 0.36^a^	1.8 ± 0.32^a^	1.82 ± 0.37^a^	1.77 ± 0.24^a^
PNOC	1.03 ± 0.13^b^	1.46 ± 0.22^a^	1.48 ± 0.2^a^	1.48 ± 0.29^a^	1.78 ± 0.16^a^	1 ± 0.13^b^	1.71 ± 0.22^a^	1.64 ± 0.37^a^	1.61 ± 0.24^a^	2.01 ± 0.19^a^	1.04 ± 0.06^b^	1.92 ± 0.29^a^	1.95 ± 0.29^a^	1.97 ± 0.16^a^	2.04 ± 0.37^a^
CCK	1.03 ± 0.08^b^	1.31 ± 0.25^ab^	1.37 ± 0.28^ab^	1.35 ± 0.13^ab^	1.55 ± 0.17^a^	0.98 ± 0.1^c^	1.44 ± 0.15^b^	1.43 ± 0.25^b^	1.4 ± 0.16^b^	1.77 ± 0.22^a^	1.04 ± 0.11^b^	1.91 ± 0.18^a^	1.92 ± 0.12^a^	1.94 ± 0.17^a^	1.96 ± 0.3^a^
CCKBR	1.01 ± 0.12	1.37 ± 0.27	1.36 ± 0.18	1.36 ± 0.24	1.42 ± 0.21	1.01 ± 0.12^b^	1.31 ± 0.17^a^	1.33 ± 0.06^a^	1.32 ± 0.1^a^	1.58 ± 0.27^a^	1.05 ± 0.09	1.55 ± 0.42	1.56 ± 0.35	1.46 ± 0.32	1.53 ± 0.25
PENK	1.01 ± 0.1	1.14 ± 0.17	1.13 ± 0.22	1.19 ± 0.33	1.25 ± 0.26	1.04 ± 0.13^b^	1.64 ± 0.28^a^	1.55 ± 0.25^a^	1.54 ± 0.28^a^	1.84 ± 0.31^a^	1.03 ± 0.11^b^	1.48 ± 0.33^ab^	1.44 ± 0.35^ab^	1.43 ± 0.27^ab^	1.63 ± 0.32^a^
PDYN	1.01 ± 0.08^b^	1.19 ± 0.09^b^	1.17 ± 0.13^b^	1.25 ± 0.14^b^	1.68 ± 0.21^a^	1.05 ± 0.15^b^	1.45 ± 0.24^ab^	1.37 ± 0.28^ab^	1.39 ± 0.09^ab^	1.71 ± 0.37^a^	1.02 ± 0.07	1.44 ± 0.46	1.42 ± 0.33	1.39 ± 0.38	1.59 ± 0.33
POMC	1.03 ± 0.1^b^	1.31 ± 0.29^ab^	1.29 ± 0.22^ab^	1.26 ± 0.23^ab^	1.56 ± 0.19^a^	1.05 ± 0.14^c^	1.59 ± 0.28^b^	1.59 ± 0.19^b^	1.58 ± 0.27^b^	2.09 ± 0.2^a^	1 ± 0.11^b^	1.2 ± 0.16^b^	1.15 ± 0.22^b^	1.32 ± 0.23^ab^	1.55 ± 0.2^a^
MOR	1.02 ± 0.09^b^	1.16 ± 0.18^b^	1.16 ± 0.11^b^	1.19 ± 0.18^ab^	1.46 ± 0.22^a^	1.02 ± 0.08^b^	1.69 ± 0.28^a^	1.64 ± 0.23^a^	1.7 ± 0.22^a^	1.82 ± 0.4^a^	1.03 ± 0.09^b^	1.32 ± 0.2^ab^	1.29 ± 0.18^ab^	1.37 ± 0.26^ab^	1.73 ± 0.42^a^
**HYPOTHALAMUS**															
Tβ4	1.02 ± 0.14^b^	1.48 ± 0.22^a^	1.51 ± 0.27^a^	1.48 ± 0.25^a^	1.16 ± 0.17^b^	1.02 ± 0.12^b^	1.67 ± 0.13^a^	1.62 ± 0.16^a^	1.64 ± 0.18^a^	1.24 ± 0.08^ab^	1.04 ± 0.1^b^	1.66 ± 0.32^a^	1.6 ± 0.34^a^	1.66 ± 0.37^a^	1.29 ± 0.35^ab^
PNOC	0.99 ± 0.1^b^	1.21 ± 0.18^ab^	1.26 ± 0.11^ab^	1.17 ± 0.27^ab^	1.41 ± 0.22^a^	1.02 ± 0.1^b^	1.59 ± 0.18^a^	1.68 ± 0.23^a^	1.68 ± 0.23^a^	1.75 ± 0.33^a^	0.99 ± 0.11^b^	1.9 ± 0.32^a^	1.87 ± 0.35^a^	1.76 ± 0.32^a^	1.72 ± 0.43^a^
CCK	1.01 ± 0.09	1.07 ± 0.17	1.07 ± 0.16	1.04 ± 0.14	1.17 ± 0.12	1.05 ± 0.13^b^	1.47 ± 0.16^a^	1.46 ± 0.15^a^	1.52 ± 0.16^a^	1.61 ± 0.25^a^	1.04 ± 0.07^b^	1.69 ± 0.34^a^	1.65 ± 0.31^a^	1.61 ± 0.34^a^	1.66 ± 0.44^a^
CCKBR	0.96 ± 0.08^b^	1.32 ± 0.18^a^	1.38 ± 0.22^a^	1.35 ± 0.09^a^	1.53 ± 0.15^a^	1.01 ± 0.12^b^	1.34 ± 0.19^ab^	1.36 ± 0.23^ab^	1.37 ± 0.35^ab^	1.51 ± 0.35^a^	1.01 ± 0.09^b^	1.66 ± 0.38^a^	1.67 ± 0.36^a^	1.61 ± 0.41^a^	1.64 ± 0.13^ab^
PENK	1.02 ± 0.13^b^	1.2 ± 0.12^b^	1.2 ± 0.12^b^	1.17 ± 0.1^b^	1.57 ± 0.19^a^	1 ± 0.11^b^	1.37 ± 0.11^a^	1.37 ± 0.13^a^	1.37 ± 0.12^a^	1.49 ± 0.27^a^	1.01 ± 0.13	1.24 ± 0.2	1.28 ± 0.2	1.22 ± 0.36	1.43 ± 0.26
PDYN	1.02 ± 0.07^b^	1.14 ± 0.14^ab^	1.11 ± 0.18^b^	1.09 ± 0.17^b^	1.4 ± 0.18^a^	1.02 ± 0.16^c^	1.59 ± 0.11^b^	1.6 ± 0.19^b^	1.6 ± 0.17^b^	2.07 ± 0.22^a^	1.04 ± 0.11	1.49 ± 0.4	1.44 ± 0.43	1.39 ± 0.36	1.62 ± 0.26
POMC	1.04 ± 0.12^b^	1.19 ± 0.24^ab^	1.18 ± 0.13^ab^	1.14 ± 0.22^b^	1.47 ± 0.19^a^	0.99 ± 0.12^b^	1.87 ± 0.24^a^	1.89 ± 0.19^a^	1.89 ± 0.18^a^	2.22 ± 0.22^a^	1.03 ± 0.12^b^	1.51 ± 0.2^ab^	1.46 ± 0.3^ab^	1.57 ± 0.35^a^	1.69 ± 0.35^a^
MOR	1.03 ± 0.11^b^	1.16 ± 0.21^b^	1.18 ± 0.12^b^	1.19 ± 0.14^b^	1.52 ± 0.25^a^	1.05 ± 0.12^c^	1.9 ± 0.16^b^	1.93 ± 0.29^b^	1.93 ± 0.16^b^	2.38 ± 0.09^a^	1.04 ± 0.11	1.24 ± 0.16	1.42 ± 0.17	1.26 ± 0.24	1.15 ± 0.42
**MIDBRAIN**															
Tβ4	1.03 ± 0.14^ab^	1.29 ± 0.21^ab^	1.29 ± 0.27^ab^	1.32 ± 0.18^a^	0.97 ± 0.13^b^	0.98 ± 0.11^b^	1.59 ± 0.28^a^	1.61 ± 0.27^a^	1.62 ± 0.27^a^	1.25 ± 0.22^b^	1.04 ± 0.14^b^	1.91 ± 0.14^a^	1.91 ± 0.14^a^	1.92 ± 0.31^a^	1.75 ± 0.16^a^
PNOC	1.02 ± 0.06^b^	1.12 ± 0.18^b^	1.1 ± 0.22^b^	1.15 ± 0.14^ab^	1.43 ± 0.22^a^	1.01 ± 0.11^b^	1.53 ± 0.18^a^	1.54 ± 0.18^a^	1.47 ± 0.27^a^	1.76 ± 0.24^a^	0.99 ± 0.09^b^	2.08 ± 0.12^a^	2.06 ± 0.15^a^	2.04 ± 0.12^a^	2.01 ± 0.26^a^
CCK	1.04 ± 0.06^b^	1.11 ± 0.2^b^	1.07 ± 0.21^b^	1.16 ± 0.08^ab^	1.48 ± 0.2^a^	1.01 ± 0.11^c^	1.69 ± 0.28^b^	1.73 ± 0.29^b^	1.78 ± 0.12^b^	1.97 ± 0.16^a^	1.03 ± 0.13^c^	2.02 ± 0.18^b^	2.2 ± 0.2^b^	2.08 ± 0.26^b^	2.18 ± 0.23^a^
CCKBR	1.06 ± 0.12	1.27 ± 0.4	1.12 ± 0.24	1.19 ± 0.26	1.45 ± 0.24	1 ± 0.09^b^	1.72 ± 0.26^a^	1.76 ± 0.21^a^	1.76 ± 0.29^a^	1.59 ± 0.41^a^	1.02 ± 0.1^c^	1.93 ± 0.24^a^	1.99 ± 0.23^a^	2.04 ± 0.11^a^	1.47 ± 0.19^b^
PENK	1.02 ± 0.11	1.09 ± 0.21	1.06 ± 0.23	1.02 ± 0.18	1.18 ± 0.18	1.06 ± 0.15^b^	1.75 ± 0.11^a^	1.79 ± 0.14^a^	1.77 ± 0.17^a^	2.01 ± 0.2^a^	1.01 ± 0.13^c^	1.41 ± 0.14*a*^b^	1.32 ± 0.15^b^	1.35 ± 0.21^b^	1.7 ± 0.2^a^
PDYN	0.99 ± 0.08^c^	1.3 ± 0.16^bc^	1.33 ± 0.2^b^	1.35 ± 0.1^b^	1.71 ± 0.32^a^	1.01 ± 0.12^c^	1.64 ± 0.26^b^	1.59 ± 0.2^b^	1.69 ± 0.3^b^	2.13 ± 0.37^a^	1 ± 0.09	1.01 ± 0.14	1.07 ± 0.38	1.19 ± 0.22	1.19 ± 0.37
POMC	1.01 ± 0.07^b^	1.18 ± 0.33^ab^	1.24 ± 0.21^ab^	1.21 ± 0.14^ab^	1.61 ± 0.36^a^	1.01 ± 0.11^b^	1.59 ± 0.32^a^	1.64 ± 0.13^a^	1.63 ± 0.36^a^	2.06 ± 0.37^a^	1.02 ± 0.07	0.99 ± 0.22	1.07 ± 0.33	1.01 ± 0.24	1.13 ± 0.22
MOR	1 ± 0.04^b^	1.18 ± 0.14^b^	1.13 ± 0.22^b^	1.18 ± 0.22^b^	1.65 ± 0.25^a^	1.03 ± 0.07^c^	1.85 ± 0.2^b^	1.83 ± 0.25^b^	1.76 ± 0.16^b^	2.23 ± 0.2^a^	1.01 ± 0.08^c^	1.51 ± 0.24^b^	1.47 ± 0.21^b^	1.57 ± 0.18^b^	1.95 ± 0.22^a^
**MEDULLA**															
Tβ4	1.01 ± 0.12	1.25 ± 0.17	1.28 ± 0.35	1.25 ± 0.22	0.95 ± 0.23	1.05 ± 0.15^b^	1.82 ± 0.17^a^	1.81 ± 0.23^a^	1.72 ± 0.28^a^	1.51 ± 0.16^a^	0.98 ± 0.11^c^	2.2 ± 0.18^a^	2.24 ± 0.28^a^	2.19 ± 0.26^a^	1.66 ± 0.26^b^
PNOC	1.02 ± 0.07	1.06 ± 0.29	1.05 ± 0.18	1.03 ± 0.21	1.1 ± 0.21	0.99 ± 0.08^b^	1.68 ± 0.28^a^	1.6 ± 0.39^a^	1.66 ± 0.14^a^	1.76 ± 0.43^a^	1.02 ± 0.09^b^	1.87 ± 0.11^a^	1.91 ± 0.13^a^	1.89 ± 0.17^a^	2.06 ± 0.24^a^
CCK	1.05 ± 0.09^b^	1.18 ± 0.15^b^	1.21 ± 0.25^b^	1.22 ± 0.19^b^	1.57 ± 0.26^a^	1.03 ± 0.07^b^	1.8 ± 0.14^a^	1.79 ± 0.29^a^	1.78 ± 0.16^a^	1.97 ± 0.09^a^	1.02 ± 0.14^b^	2.06 ± 0.13^a^	1.93 ± 0.25^a^	1.95 ± 0.15^a^	2.16 ± 0.19^a^
CCKBR	0.98 ± 0.06	1.11 ± 0.2	0.98 ± 0.2	0.98 ± 0.27	1.29 ± 0.22	1.03 ± 0.13^b^	1.62 ± 0.29^a^	1.61 ± 0.29^a^	1.61 ± 0.33^a^	1.95 ± 0.16^a^	1.05 ± 0.13^b^	1.96 ± 0.26^a^	1.97 ± 0.57^a^	1.82 ± 0.31^a^	2.14 ± 0.33^a^
PENK	1.02 ± 0.09	1.22 ± 0.31	1.19 ± 0.33	1.15 ± 0.36	1.11 ± 0.24	1.01 ± 0.11^b^	1.54 ± 0.15^ab^	1.42 ± 0.33^ab^	1.42 ± 0.34^ab^	1.82 ± 0.34^a^	1.01 ± 0.07	1.01 ± 0.2	1.03 ± 0.18	0.96 ± 0.24	1.29 ± 0.27
PDYN	1.02 ± 0.13^b^	0.99 ± 0.18^b^	1.09 ± 0.1^b^	1.06 ± 0.21^b^	1.42 ± 0.17^a^	1.04 ± 0.12^b^	1.39 ± 0.29^b^	1.38 ± 0.2^b^	1.41 ± 0.28^b^	1.86 ± 0.27^a^	1.03 ± 0.12^b^	0.97 ± 0.13^b^	0.97 ± 0.25^b^	1.04 ± 0.33^b^	1.42 ± 0.1^a^
POMC	1.02 ± 0.09^b^	1.22 ± 0.24^ab^	1.24 ± 0.34^ab^	1.28 ± 0.25^ab^	1.54 ± 0.34^a^	0.96 ± 0.11^c^	1.47 ± 0.26^b^	1.44 ± 0.25^b^	1.49 ± 0.33^b^	2.05 ± 0.14^a^	0.99 ± 0.1	0.97 ± 0.34	0.95 ± 0.24	1.09 ± 0.34	1.36 ± 0.27
MOR	1.02 ± 0.11^b^	1.44 ± 0.14^a^	1.45 ± 0.08^a^	1.44 ± 0.18^a^	1.55 ± 0.17^a^	1 ± 0.11^c^	1.49 ± 0.32^b^	1.47 ± 0.16^b^	1.41 ± 0.1^b^	1.89 ± 0.29^a^	1.06 ± 0.1^c^	1.52 ± 0.21^ab^	1.42 ± 0.32^bc^	1.45 ± 0.23^bc^	1.92 ± 0.23^a^

*The measured brain areas included hypothalamus, thalamus, cortex, midbrain and medulla. The mRNA expressions of Tβ4, thymosin beta 4; PONC, prepronociceptin; CCK, cholecystokinin; CCKBR,CCK B receptor; POMC, proopiomelanocortin; PENK, proenkephalin; PDYN, prodynorphin; MOR, mu opioid receptor were measured with qPCR. The values with different letters (a-c) differ significantly in different groups at the same day (P < 0.05). One-way ANOVA followed by Bonferroni's post-test*.

In cortex, EA treatment induced an increase in Tβ4, CCK, and MOR mRNAs (day 1–8), PNOC and POMC mRNAs (day 4 and 8), PENK and PDYN mRNAs (day 4), and CCKBR mRNA (day 8) compared with sham treatment. EA-Tβ4 siRNA treatment caused a decrease in Tβ4 mRNA (day 1–8), but an increase PDYN and MOR mRNAs (day 1 and 4) and PENK mRNA (day 4) compared with EA treatment.

In thalamus, compared with rats in Sh-EA group, rats in EA-tr group showed increased Tβ4 and PNOC (day 1–8), CCK (day 4 and 8), and CCKBR, PENK, POMC, and MOR (day 4) mRNAs. Compared with rats in EA-tr group, rats in EA-Tβ4-si group showed decreased Tβ4 mRNA (day 1 and 4), but increased CCK (day 4), POMC (day 4 and 8), and PDYN and MOR (day 1) mRNAs.

In hypothalamus, mRNA levels of Tβ4 (day 1–8), PNOC and CCK (day 4 and 8), CCKBR (day 1 and 8), and PENK, PDYN, POMC, and MOR (day 4) mRNAs in EA-tr group were higher than those in Sh-EA group. EA-Tβ4 siRNA treatment induced a decrease in Tβ4 (day 1), but an increase in PENK (day 1), and PDYN and MOR (day 1 and 4) mRNAs compared with EA treatment.

In midbrain, EA-treated rats exhibited higher Tβ4, PNOC, CCK, CCKBR, PENK, and MOR (day 4 and 8), PDYN (day 1 and 4), and POMC (day 4) mRNAs than sham-treated rats. EA-Tβ4 siRNA-treated rats showed decreased Tβ4 (day 4), PNOC (day 1), CCK, and PDYN (day 1 and 4), CCKBR and PENK (day 8) and MOR (day 1–8) mRNAs compared with those in EA-treated rats.

In medulla, EA treatment caused an increase in Tβ4, PNOC, CCK and CCKBR mRNAs (day 4 and 8), in POMC mRNA (day 4) and MOR mRNA (day 1 to 4) compared with sham treatment. EA-Tβ4 siRNA treatment induced decreased Tβ4 (day 8), CCK (day 1), PDYN (day 1–8), POMC (day 4), and MOR (day 4 and 8) mRNAs compared with EA treatment.

## Discussion

EA has been extensively used for treating various diseases (Liu et al., 2009; Shah et al., 2016; Cui et al., 2017b; Wan et al., 2018), especially pain disorders (Zeng et al., 2016; Hu et al., 2017; Wan et al., 2017). However, it also can provoke EAT, which is a negative effect for its application. Some researchers use different EA modalities to establish EAT. Tian et al. (1998) used 100 Hz EA to stimulate rats for consecutive 6 h, and found that the pain threshold decreased at 1 h (105%) and approximated to the level before EA (25%) at 6 h, suggesting that continuous EA can provoke a rapid EAT. Repeated EA is more commonly used than continuous EA for pain therapy in clinical practice. Cui et al. (2016, 2017a) stimulated rats with 2/15 Hz and 2 Hz EA (30 min/day, total 8 days), and found that the pain thresholds decreased from 59.6 ± 4.6% and 61.4 ± 5.5% at day 1–2.1 ± 4.1% and 1.9 ± 7.4% at day 8, respectively, showing that repeated EA can elicit chronic EAT. Wang et al. (2002) used 2 Hz EA for 30 min every time for total 6 times with different intervals (0, 1, 2, and 3 days, respectively) in rats, and found that EA with the interval of 1 day induced EAT whereas EA with the interval of 3 days produced potent analgesia, showing that EA interval is an important factor influencing the development of EAT. In the present study, 2/15 Hz was used to stimulate rats for 30 min once daily for consecutive 8 day. The TFL change rate was reduced from 47.0 ± 3.4% at day 1 to 1.5 ± 1.5% at day 8, indicating the formation of EAT. The result in our experiment is similar to the report by Cui et al. (2016, 2017a).

Intracerebroventricular injection has been frequently used to study neural substrates in acupuncture analgesia (Tang et al., 1997; Cui et al., 2017a). Icv injection of antibody is a classical method for verifying the function of neural substrates. SiRNA has been shown to be a powerful technology allowing the silencing of mammalian genes with great specificity and potency (Alisky and Davidson, 2004; Aigner, 2006). A number of experiments have demonstrated the potential of appropriately designed siRNAs (Karagiannis and Elosta, 2005). Several formulations, including liposomal and viral vectors, have been shown to be efficacious for delivering siRNAs to local sites of the CNS (Bumcrot et al., 2006). Intracereboventricularly injected siRNA can maintain a longer effect because the cerebrospinal fluid lacks a significant nuclease (Guo et al., 1996). In the present study, strong fluorescence at 24 h after icv injection of the mixture of fluorescence-conjugated siRNA and lipofection was observed in several brain areas, including hypothalamus, thalamus, cortex, midbrain and medulla, which implied that siRNA was successfully transfected into the cells in targeted brain tissues via the cerebrospinal fluid. The dosage (5 μL Tβ4 siRNA mixture with 10 μL lipofection) and a single application of the mixture solution were determined according to fluorescence intensity in brain areas and the duration of Tβ4 siRNA effect (a lower level of Tβ4 lasted for 8 days), respectively, in our pretest.

Numerous studies confirmed that EOPs in the CNS were main mediators to participate in EA analgesia regulation (Han et al., 1984; Han, 2003). Hence, opioid tolerance may be an important mechanism for EAT. In the present study, EOPs levels in the measured areas were positively correlated with the change rates in TFLs of rats treated with repeated EA.Tβ4 siRNA injection evoked an increase in ENK, DYN and END levels in most measured areas. ENK, DYN, or END level was negatively correlated with Tβ4 levels in the measured areas except in medulla where no correlation was observed between ENK and Tβ4 level. These results obviously displayed that the inhibition of Tβ4 partly reversed the expression of EOPs, hereby delayed the formation of EAT.

It has been found that EA induces the release of anti-opioid peptides (CCK-8 and OFQ) as it increases levels of opioid peptides in the CNS (Bian et al., 1993; Tian et al., 1998). Therefore, the roles of these anti-opioid peptides in EAT have attracted more attention. Han et al. (1985) injected CCK-8 into the cerebroventricle or spinal subarachnoid space in rats, and found that CCK-8 dose-dependently antagonized EA-induced analgesic effect. They also confirmed that icv or intrathecal injection of the antiserum against CCK-8 postponed or reversed the EAT (Han et al., 1986). Microinjection of OFQ into rats' periaqueductal gray remarkably antagonized EA analgesia in a dose-related manner (Zhi-Qi, 2008). Tian et al. (1998) reported that icv injection of OFQ antibody reversed EAT. These studies indicate that endogenous CCK-8 and OFQ participate in the formation of EAT. In this trial, Tβ4 siRNA treatment caused unchanged OFQ and CCK-8 levels in most brain areas except midbrain and hypothalamus. Studies found that the increased CCK-8 and OFQ were along with the increment of EOPs, which is believed to be a negative feedback of the former (Huang et al., 2003; Wang et al., 2006). Therefore, the increase of CCK-8 and OFQ in midbrain and hypothalamus may be also due to negative feedback on increased opioid peptides induced by Tβ4 siRNA.

Since opioid and anti-opioid peptides exert biological functions through their receptors, the roles of opioid and anti-opioid receptors in the development of EAT appeal to researchers. MOR, one of classic opioid receptors with a high affinity for ENK and END, is involved in EA-induced analgesia (Han et al., 1981a; Chen and Han, 1992a; Huang et al., 2000). CCK-8 could interact with CCK receptors (especially CCKBR) in the specific sites of the CNS to interfere with the functions of MOR, resulting in anti-analgesic effect (Wang et al., 1990; Shen et al., 1995; Huang et al., 2007). MOR and CCKBR can be selected as representatives of EOP- and anti-EOP- receptors, respectively. Ni et al. (1987) found that repeated EA resulted in EAT and decreased opioid receptors in rats' brain. Huang et al. (2007) demonstrated that CCKBR antagonist (L365, 260) potentiated 100 Hz EA-induced analgesia and reversed chronic tolerance to 100 Hz EA in mice. These findings demonstrated that MOR and CCKBR were involved in EAT. In the current study, the level of MOR decreased as EA application frequencies increased, which was consistent with the report of Ni et al. (1987). However, Tβ4 siRNA treatments caused increased MOR level; MOR levels were negative correlation with Tβ4 levels in the measured brain areas. Tβ4 siRNA caused CCKBR levels to be unchanged in thalamus and midbrain, and to be increased in cortex, hypothalamus and medulla at some specific time points. However, CCKBR levels were not correlated with Tβ4 levels. These results indicated that Tβ4 facilitated EAT probably via influencing opioid receptors. However, the underlying mechanisms need to be further studied.

Tβ4 is a small molecule polypeptide that widely exists in the CNS. Tβ4 can be released from neurons, but the targets it works on are unclear. Xiong et al. (2012a,b) reported that Tβ4 provided neuro-protection and neuro-restoration in experimental traumatic brain injury. Santra et al. (2012) reported that Tβ4 mediated oligodendrocyte differentiation via the inhibition of p38MAPK and the reduction of phosphorylated JNK accumulation. Jeon et al. (2013) found that Tβ4 facilitated mesenchymal stem cells proliferation through activation of ERK. These studies suggested that Tβ4 exerted neuro-protection via MAPK (ERK1/2, P38, and JNK) signal pathways. (Chen and Sommer, 2009) reported that MAPK signaling pathways were activated in the CNS and the periphery in the chronic morphine tolerance which was reduced by the inhibitors of these pathways. The similarity between EA and morphine tolerance has been demonstrated in a cross-tolerance study (Han et al., 1981b). Cui et al. (2017a) reported that the MAPK signaling pathway was targeted by miRNAs involved in chronic EAT. The previous studies suggest that there is a potential relationship between MAPK signal pathways and chronic EAT (Han et al., 1981b; Cui et al., 2017a). However, whether Tβ4 activates these pathways in chronic EAT needs to be confirmed.

In summary, repeated EA can result in decreased TFL, which is a negative effect of its treatment. Studies have showed that Tβ4 has neuroprotective and neuro-nutritional effects. Repeated EA, as a stimulus, caused increased Tβ4 in brain areas. Tβ4 siRNA icv injection postponed the formation of EAT, reversed decreased opioid- and their receptors induced by EAT in most brain areas. These results confirmed Tβ4 facilitated EAT probably through negatively changing endogenous opioid peptides and their receptors and positively influencing anti-opioid peptides in the CNS. Neuromodulators (opioid peptides and non-opioid peptides) in the CNS are distributed in different regions in which the neurons communicate with each other through neural fibers, and constitute a regulating network. EA induces the release of the neuromodulators in the specific regions, thereby exerts the analgesic modification. In the present study, the expression levels of opioid- and anti-opioid-peptides and their receptors induced by Tβ4 siRNA were fluctuated in some brain areas, indicating the complex regulations of these neuromodulators and brain areas in the formation of EAT. Although the present study exhibited that Tβ4 siRNA delayed the formation of EAT through affecting the expression of opioid peptides, anti-opioid peptides and opioid receptors in a wide range of the brain regions. Since substances are not evenly distributed in the specific pain-regulated nuclei, the brain area sampling may result in their homogenization; it may not accurately reflect their levels in the specific nuclei. It will be more important to investigate the effect of Tβ4 on neuromodulators in the specific nuclei in the formation of EAT. In addition, the role of Tβ4 in anti-analgesic neural circuits or cellular signaling pathways needs to be further studied. Anyhow, our results provided an important foundation for investigating the molecular mechanisms by which Tβ4 modifies EAT in the future.

## Author Contributions

MD contributed to the conception and design of the study. JW, YD, QZ, SN, JS, and CS performed animal experiments, collected samples and accomplished the laboratory investigations. YD, JW, and SN performed the acquisition of data. YD, JW, and QZ conducted the data analysis and interpretation of data. WJ drafted the manuscript. MD, YD, and JW revised the manuscript. All authors read and approved the final manuscript.

### Conflict of Interest Statement

The authors declare that the research was conducted in the absence of any commercial or financial relationships that could be construed as a potential conflict of interest.

## Abbreviations

CCK-8: Cholecystokinin octapeptide
CCKBR: CCK B type receptor
CNS: Central nerve system
DYN: Dynorphin
EA: Electroacupuncture
EAT: EA tolerance
EOP: Endogenous opioid peptides
ENK: Enkephalin
END: Endorphin
icv: Intracerebroventricular
MOR: Mu opioid receptor
OFQ: Orphanin FQ
PONC: Prepronociceptin
PDYN: Prodynorphin
PENK: Proenkephalin
POMC: Proopiomelanocortin
TFL: Tail flick latency
Tβ4: Thymosin beta 4.
